# Bacterial resistance to antimicrobial agents: selected problems in France, 1996 to 1998.

**DOI:** 10.3201/eid0503.990301

**Published:** 1999

**Authors:** H Aubry-Damon, P Courvalin


### Bacterial Resistance to Antimicrobial Agents: Selected Problems in France, 1996 to 1998

**Helene Aubry-Damon* and Patrice Courvalin†**
*Institut de Veille Sanitaire, Saint-Maurice, France; and
†Centre National de Référence des Antibiotiques, Institut Pasteur, Paris, France

Dr. Aubry-Damon is a microbiologist specializing in antimicrobial resistance at the National Institute for Public Health Surveillance, Saint-Maurice, France. The institute has recently carried out an exploratory study of a national program for surveillance of antimicrobial resistance in France. Dr. Aubry-Damon also collaborates closely with the French National Reference Center for Antibiotics. (No photo available)

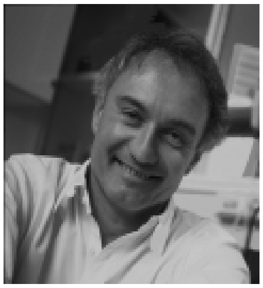

Dr. Courvalin is professor at the Institut Pasteur, where he directs the French National Reference Center for Antibiotics and heads the Antibacterial Agents Unit. Dr. Courvalin specializes in the genetics and biochemistry of antibiotic resistance. His research has led to revision of the description of the natural dissemination of antibiotic resistance genes. His laboratory demonstrated that pathogenic bacteria can promiscuously exchange genetic material conferring antibiotic resistance, documented that conjugation could account for dissemination of resistance determinants between phylogenetically remote bacterial genera, elucidated the transposition mechanism of conjugative transposons from gram-positive cocci, and recently, obtained direct gene transfer from bacteria to mammalian cells.

Surveillance of antibiotic resistance in human pathogens has long been performed in France. Existing surveillance relies on national reference centers dedicated to various bacterial genera and on networks of volunteer medical microbiologists, mainly in general hospitals but also in private laboratories. Regional data (often initiated at the request of and funded by the pharmaceutical industry) have been available since the early 1950s. Because of the major health problems caused by antibiotic resistance in the last few years, attempts have been made to organize a national surveillance program similar to those being established in other European countries. Although this update reviews recent data from France, the representativeness of the data has not been assessed. In addition, these are raw data, and their clinical importance remains to be seen; for example, the contribution of bacterial isolates to infection or colonization is, in most instances, unknown.

Address for correspondence: P. Courvalin, Unité des Agents Antibactériens, Institut Pasteur, 28, rue du Dr. Roux, 75724 Paris Cedex 15, France; fax: 33-1-45-68-83-19; e-mail: pcourval@pasteur.fr.

### Evolution of Antibiotic Resistance in Hospitals

### Dissemination of β-Lactamases in Gram-Negative Bacilli

In France, β-lactamases and fluoroquinolones are the most frequently prescribed antibiotics in *Enterobacteriaceae* infections. A multicenter study (14 hospitals) across the country analyzed the antibiotic susceptibility of 2,507 and 2,312 consecutive, nonrepetitive enterobacteria responsible for infection in 1996 and 1997, respectively (1,2). Strains were isolated from inpatients (86%) in intensive care (ICU) (12%), surgical (17%), medical (37%), and geriatric (9%) units. The majority of isolates were from urine (71%), pus (9%), and bronchopulmonary specimens (8%). *Escherichia coli* (64%) was isolated most frequently, mainly in outpatients, whereas *Klebsiella* spp. and *Enterobacteriaceae* with inducible β-lactamases predominated in ICUs. Resistance of *E. coli* to amoxicillin and cefotaxime was 47% and 0.5%, respectively. In 1997, the frequency of isolates producing an extended-spectrum β-lactamase varied by species: in *Enterobacter aerogenes,* 56%; in *Klebsiella pneumoniae,* 15%; and in *E. coli,* 0.5%. The incidence differed within and between hospitals. Such strains arise in response to the selective pressure exerted by use of extended-spectrum cephalosporins (3); infections with such strains have also been associated with hospitalization in ICUs. Production of β-lactamases resistant to β-lactam-enzyme inhibitor combinations in *E. coli* was approximately 3.5% (2). Susceptibility to fluoroquinolones was high (66%-97% ciprofloxacin-susceptible) except in *E. aerogenes* and *Serratia marcescens* (35%-52% ciprofloxacin-susceptible) (1).

The organisms most frequently isolated in ICUs in 1995 belonged to the family *Enterobacteriaceae* (59%) and *Pseudomonas aeruginosa* (25%) (4). In 1997, 1,362 nonrepetitive *P. aeruginosa* (5% of all clinical isolates) were collected in 13 teaching hospitals (5). The lowest rates of susceptibility to ceftazidime (<75%), amikacin (70%), and ciprofloxacin (65%) were observed with serotype 12 (the fourth main serotype). Among penicillinase-producing strains, the percentages of resistance to amikacin and ciprofloxacin were 80% and 93%, respectively; these figures were substantially higher in β-lactam-resistant *P. aeruginosa* than in susceptible strains.

### Spread of Methicillin-Resistant and Gentamicin-Susceptible *Staphylococcus aureus*

Methicillin-resistant *S. aureus* (MRSA) is one of the most frequent nosocomial pathogens in France as in the rest of the world. Surveys conducted in hospitals in Paris and surroundings found that MRSA decreased from 42% in 1992 to 37% in 1997 and that the incidence of MRSA colonization-infection (approximately 0.65 per 100 admissions) also decreased after national recommendations against dissemination of multidrug-resistant bacteria were implemented (6,7). However, a survey of 26 geographically representative hospitals found that the incidence of gentamicin-susceptible MRSA progressively increased because of the presence of a predominant clone (H. Lelièvre, G. Lina, M.E. Jones, et al., unpub. obs.). The epidemiologic situation in France is complex. The endemic aminoglycoside-resistant MRSA strain persisted while new clones became endemic in hospitals, perhaps after changes in the use of aminoglycosides (decrease of gentamicin and increase of amikacin consumption) (8). The first vancomycin-intermediate *S. aureus* was isolated in a French hospital in 1995; no other cases of MRSA with reduced susceptibility to vancomycin have been reported (9).

### Most Frequent Macrolide-Resistance Mechanisms among Staphylococci

Over 3 weeks in 1995, 607 staphylococci were collected in 32 hospitals (10). Of these, 45.5% of the *S. aureus* and 54% of the coagulase-negative staphylococci were resistant to methicillin, and 71.5% of MRSA were resistant to macrolides. Of these MRSA strains, 75% were constitutively resistant, whereas 76% of MSSA were inducibly resistant. A similar distribution (61% vs. 27.5%) was observed among coagulase-negative staphylococci. Resistance to at least one of the macrolide, lincosamide, and streptogramin antibiotics (88%) was due to the presence of the *ermA* and *ermC* genes, which confer resistance by modifying the ribosomal target. The *ermA* gene was more common in MRSA (57.6%) than in MSSA (5.6%), where *ermC* was predominant (20.1%). *ermC* was also common among methicillin-susceptible coagulase-negative staphylococci (14%). Only a few strains had the *ermB* gene, which is found in animal strains. Macrolide resistance by efflux due to acquisition of the *msrA* gene was more prevalent in coagulase-negative staphylococci (14.6%) than in *S. aureus* (2.1%). The incidence of lincomycin-resistant but macrolide- and streptogramin-susceptible staphylococci was low: 0.2% in *S. aureus* and 4.6% in *Staphylococcus epidermidis* (11). The prevalence of pristinamycin-resistant (and also most probably quinupristin/dalfopristin-resistant) strains remained low because of the low incidence of resistance to streptogramins type A (pristinamycin has limited use in France).

### Dissemination of Resistance in the Community

### Effect of Antibiotics on Oropharyngeal Flora

#### Antibiotic Resistance in *Streptococcus pneumoniae*

The National Reference Center for Pneumococci determined the susceptibility to antibiotics of 2,837 *S. pneumoniae* isolated in 1997. The incidence of *S. pneumoniae* with reduced susceptibility to penicillin G increased from 3.8% in 1987 to 48% in 1997 (12). Whereas 53% of all strains were resistant to macrolides, 80% of penicillin-resistant strains were macrolide-resistant; 15% of all strains (versus 51% of penicillin-resistant strains) were resistant to tetracycline, and 10% (versus 66%), respectively, were resistant to trimethoprim-sulfamethoxazole (F. Goldstein et al., unpub. data). According to a 1997 survey of 18 regional laboratories in France (11,757 strains collected), 27% had intermediate levels of resistance to penicillin G, and 13.5% were fully resistant. The rates varied considerably by region (highest in southwest and central France), age (highest [37.4% penicillin G-intermediate and 21.5% resistant] in children <16 years old), and specimen source (highest in middle ear and sinus specimens) (13).

### Resistance to Macrolides in β-Hemolytic Streptococci

In 1995, a national survey in 98 hospitals of invasive infections due to *Streptococcus pyogenes* found that 5.2% to 9.8% of the strains isolated from blood were erythromycin-resistant (A. Bouvet, pers. comm.) (14).

### Vaccination against and Resistance in *Haemophilus influenzae* Type b

To monitor the trends in *H. influenzae* meningitidis and the prevalence of resistance, the National Reference Center conducted a survey of approximately 80 hospitals (15). Since vaccination for Hib invasive infections began in 1993, the percentage of capsulated isolates has decreased 5% per year. Moreover, resistance to antimicrobial drugs decreased among Hib and increased among noncapsulated strains isolated from upper and lower respiratory tract infections. The percentage of β-lactamase-producing *H. influenzae* increased progressively from 22% in 1992 to 35% in 1997, with a similar evolution for kanamycin resistance. Tetracycline and chloramphenicol resistance remained stable in 1997—less than 10% and 2%, respectively (15,16).

### Antibiotic Resistance in *Neisseria meningitidis*

Meningococcal resistance to antibiotics is emerging in France. The incidence of *N. meningitidis* with reduced susceptibility to penicillin G (MICs from 0.125 mg/L to 1 mg/L) increased from less than 1% in 1991 to 18% in 1996 (17,18). The strains belonged to various serogroups; most belonged to serogroup B, none produced a β-lactamase, and all were susceptible to cefotaxime and ceftriaxone. Resistance to rifampin, used for prophylaxis of secondary cases in France, remained low (0.02% in 1996).

### Effect of Antibiotics on Digestive Flora

### Antimicrobial Resistance in *Helicobacter pylori*

Susceptibility testing of *H. pylori* from 535 patients with a positive CLO test was performed in 1997 (19). Depending on the method, the percentages of clarithromycin resistance (disk-agar diffusion or MIC determination by agar dilution) and metronidazole resistance (breakpoint method at 8 mg/L or MIC determination) varied from 14.3% (95% confidence interval [CI] 11.5-17.6) to 14.0% (95% CI 11.2-17.3) and from 30.5% (95% CI 25.6-34.5) to 23.6% (95% CI 20.1-27.5), for the two antibiotics, respectively. No resistance to amoxicillin was observed.

### Fluoroquinolone Resistance in *Campylobacter* and *Salmonella* Hadar

The evolution of antimicrobial resistance in *Campylobacter jejuni* and *C. coli* is worrisome. Between 1986 and 1997, 2,713 strains of *C. jejuni* (68% of total Campylobacter isolates) were isolated from stool (94%) and blood (4%) and studied (20). Between 1993 and 1997, fluoroquinolone resistance increased from 7.4% to 32% in *C. jejuni* and from 11.8% to 52% in *C. coli.* The high resistance rate to quinolones makes them ineffective in therapy of *Campylobacter* infections. These resistance rates are similar to those in other countries (e.g., Spain, the United Kingdom) (21,22). However, the prevalence of macrolide-resistant strains remains low (3.6%). The high incidence of multidrug-resistant *Salmonella* Typhimurium DT104 (12 atypical), with 82% resistance to ampicillin, streptomycin, sulphonamide, tetracycline, and chloramphenicol, is the most serious epidemiologic problem of the last decade in France (23). The incidence of *Salmonella* Hadar is increasing, and the percentages of amoxicillin- and fluoroquinolone-resistant strains in 1997 were 72% and 75%, respectively. Fluoroquinolone resistance had not been observed before 1987 in France, Spain, and the United Kingdom. This was before concomitant introduction of ciprofloxacin into clinical use and enrofloxacin into veterinary use (in particular in the poultry industry) in the late 1980s. More than 50% of *C. jejuni* and *S.* Hadar, the most frequent serotype associated with poultry, are now fluoroquinolone-resistant in these countries.

The situation is different in Sweden, where fluoroquinolones are not readily available. Therefore, guidelines for the prudent use of antibiotics (in prophylaxis or therapy) should be developed that respect the indigenous flora of humans and animals.

### New Types of Resistance in Enterococci

The increase in the incidence of glycopeptide-resistant enterococci (GRE) isolated from hospitalized patients throughout the United States has not been observed in France. A multicenter study in 1993 showed a very low incidence of GRE: 0.2% among 251 enterococcal clinical isolates and 7.5% among *Enterococcus faecium* (24). Study of 24 ICUs in 1994 determined that the prevalence of GRE colonization in patients' fecal flora was approximately 2%, 30% of which had been present at admission. No nosocomial infection due to GRE was observed (25). GRE have been identified in human food of animal origin (40% of GRE were isolated from uncooked meat) in a French study conducted in military cafeterias in 1997 (26). Thus, food may represent a major source of human colonization with GRE in France. GRE strains isolated in France were also resistant to ampicillin, tetracycline, and macrolides. However, the percentage of high resistance levels to gentamicin among GRE was comparable to that among glycopeptide-susceptible enterococci.

### Antibiotic Resistance in *Bacteroides fragilis*

Studies of antibiotic resistance in anaerobic pathogens indicate stability of resistance to carbapenems (imipenem) and nitroimidazole antibiotics (27,28). In 1998, fewer than 2% of all *B. fragilis* from 39 hospitals were resistant to metronidazole (MICs >8 mg/L), and the number of imipenem-resistant strains remained low. However, this gene reservoir requires surveillance of resistance in *B. fragilis* infections because of the use of these antibiotics in therapy.

### Other Bacteria

### Antibiotic Resistance in *Neisseria gonorrhoeae*

The number of *N. gonorrhoeae* strains identified by the National Reference Centre for Sexually Transmitted Diseases fell sharply from 1986 to 1990 (by 81%) and more slowly from 1990 until 1999 (by 55%) (29). The number of anorectal gonococcal infections reached a plateau from 1995 to 1997 but increased again in 1998, mostly in the Paris/Ile-de-France region (V. Goulet, P. Sednaoui, et al., unpub. data). An increasing percentage of *N. gonorrhoeae* displayed diminished sensitivity to penicillin G and to tetracycline. In 1997, 15% and 30% of *N. gonorrhoeae* were resistant to penicillin G (MIC $\geq$ 2 mg/L) and tetracycline (MIC 2 $\geq$ mg/L and < 16 mg/L), respectively, by chromosomal mutation. In contrast, the percentage of strains with plasmid-mediated resistance to penicillins and tetracycline has remained stable at approximately 15% since 1994. No ceftriaxone, spectinomycin, or ciprofloxacin resistance was found until 1997, when the first ciprofloxacin-resistant strains (MIC=1 mg/L) were isolated.

### Conclusions

Antibiotic resistance trends in France are for the most part similar to trends in other European countries but with some peculiarities. For instance, fluoroquinolone resistance in *Salmonella* spp. and *Campylobacter* spp. is a problem throughout Europe. However, methicillin resistance in *Staphylococcus* is more common in France than in the Scandinavian countries, although it has started to decrease because of reinforcement of hygiene measures since 1992. Also, heavy use of 16-membered macrolides has selected for resistance in gram-positive cocci by ribosomal modification rather than by efflux. In pneumococci, decreased susceptibility to penicillins is as common in France as in Spain, but the incidence of resistance to macrolides is the highest in Europe. The public health problems caused in France by bacterial resistance to antibiotics are clearly distinct from those in North America. The incidence of enterobacteria producing extended-spectrum β-lactamases and glycopeptide-resistant enterococci remains rather low in France, as in most other European countries. In the United States, the high incidence of nosocomial GRE infections is probably caused by the heavy nosocomial use of vancomycin, particularly in hematology wards and for the prevention of colitis due to *Clostridium difficile*. In contrast, no intestinal carriage of such strains is found in the general population. The situation in Europe mirrors that in the United States. In Europe, the prevalence of nosocomial GRE infections remains low, but colonization of the population is substantial, possibly because of the use of a vancomycinlike antibiotic (avoparcin) as an animal food additive. This example stresses the need for a multidisciplinary approach to surveillance of bacterial resistance to antibiotics.

### References

1. Chardon H, Nicolas-Chanoine MH, Sirot J, and le Groupe d'Etude Multicentrique. Evaluation de la sensibilité des *Enterobacteriaceae* aux b-lactamines et aux fluoroquinolones: Résultats d'une enquête multicentrique en 1996 et 1997. Proceedings of the 18th Interdisciplinary Meeting on Anti-Infectious Chemotherapy; 1998 Dec 3-4; Paris, France. p. 129.
2. Nicolas-Chanoine MH, Sirot J, and le Groupe d'Etude Multicentrique. Caractérisation et distribution des mécanismes de résistance aux b-lactamines parmi les entérobactéries: résultats d'une enquête multicentrique en 1996. Proceedings of the 17th Interdisciplinary Meeting on Anti-Infectious Chemotherapy; 1997 Dec 4-5; Paris, France. p. 251.
3. Brun-Buisson C, Legrand P, Philippon A, Montravers F, Ansquer H, Duval J, et al. Transferable enzymatic resistance to third-generation cephalosporins during nosocomial outbreak of multiresistant *Klebsiella pneumoniae*. Lancet 1987;2:302-6.
4. Jarlier V, Fosse T, Philippon A, for the ICU Study Group. Antibiotic susceptibility in aerobic gram-positive bacilli isolated in intensive care units in 39 French teaching hospitals (ICU study). Intensive Care Med 1996;22:1057-65.
5. Cavallo JD, Leblanc F, Thabaut A, Groupe d'Etude de la Résistance de *P. aeruginosa* aux ß lactamines. Susceptibility of *Pseudomonas aeruginosa* to nine antimicrobials: a 1997 French multicenter hospital survey. Proceedings of the 38th Interscience Conference on Antimicrobial Agents and Chemotherapy; 1998 Sep 24-27; San Diego, California. Washington: American Society for Microbiology; 1998. p. 191.
6. The College de Bacteriologie-Virologie-Hygiene du Centre Hospitalier Universitaire de Paris. Surveillance des staphylocoques dorés et klebsielles multirésistants à l'Assistance Publique-Hôpitaux de Paris, 1993-1996. Bulletin Epidémiologique Hebdomadaire 1998;10:41-3.
7. Reseau de Microbiologie du C.CLIN Paris Nord et le Groupe de Microbiologistes d'Ile-de-France. Surveillance des bactéries multirésistantes a partir du laboratoire. Bulletin du Centre de Coordination de la Lutte contre les Infections Nosocomiales, Paris-Nord 1998;11:4-5.
8. Aubry-Damon H, Legrand P, Brun-Buisson C, Astier A, Soussy CJ, Leclercq R. Reemergence of gentamicin-susceptible strains of methicillin-resistant *Staphylococcus aureus*: role of an infection control program and changes in aminoglycoside use. Clin Infect Dis 1998;25:647-53.
9. Ploy MC, Grelaud C, Martin C, de Lumley L, Denis F. First clinical isolate of vancomycin-intermediate *Staphylococcus aureus* in French hospital. Lancet 1998;351:1212.
10. Lina G, Quaglia A, Reverdy ME, Leclercq R, Vandenesch F, Etienne J. Distribution of genes encoding resistance to macrolides, lincosamides and streptogramins among staphylococci. Antimicrob Agents Chemother. In press 1999.
11. Leclercq R, Brisson-Noel A, Duval J, Courvalin P. Phenotypic expression and gene heterogeneity of lincosamide inactivation in *Staphyloccus* spp. Antimicrob Agents Chemother 1991;31:1887-91.
12. Geslin P. National Reference Center for Pneumococci. France, Final Activity Report 1997.
13. Roussel-Delvallez M, Weber M, Maugein J, Thierry J, Laurans G, Fosse T, et al. Résistance du pneumocoque aux antibiotiques en 1997: résultats de 18 observatoires régionaux. Bulletin Epidémiologique Annuel 1998 report. France: National Institute for Public Health Surveillance. In press 1999.
14. Varon E, Havlickova H, Pitman C, Sarr A, Muller-Alouf H, Coignard S, et al. Comparison of invasive (septicemic) and non invasive strains of group A streptococci isolated during a one-year national survey in France. Adv Exp Med Biol 1997;418:83-5.
15. Dabernat H. Donnees de surveillance du Centre National de Référence des *Haemophilus influenzae*: avant et apres la vaccination fr. Bulletin Epidémiologique Annuel 1998 report. France: National Institute for Public Health Surveillance. In press 1999.
16. Dabernat H, Delmas C. Activité du Centre National de Référence des *Haemophilus influenzae*, années 1996-1997: le déclin du type b. Medecine et Maladies Infectieuses 1998;28:940-6.
17. Guibourdenche M, Lambert T, Courvalin P, Riou JY. Epidemiological survey of *Neisseria meningitidis* susceptibility to penicillin G in France. Pathol Biol 1997;45:729-36.
18. Struillou L, Chamoux C, Berranger C, Chouillet AM, Riou JY, Raffi F. Rapid emergence of meningococci with reduced susceptibility to penicillin in France: the need for vigilance in meningitidis treatment. Clin Microbiol Infect 1998;4:661-2.
19. Broutet N, Guillon F, Sauty E, Lethuaire D, Megraud F. Survey of the in vitro susceptibility of *Helicobacter pylori* to antibiotics in France. Gut 1998;43:All.
20. Megraud F. Les infections a Campylobacter en France 1986-1997, le Centre National de Référence des infections à Campylobacter. Bulletin Epidémiologique Annuel 1998 report. France: National Institute for Public Health Surveillance. In press 1999.
21. Gaunt PN, Piddock LJV. Ciprofloxacin-resistant *Campylobacter* spp. in humans—an epidemiologic and laboratory study. J Antimicrob Chemother 1996;37:747-57.
22. Reina J, Alomar P. Fluoroquinolone resistance in thermophilic Campylobacter spp. Lancet 1990;336:186.
23. Breuil J, Armand-Lefevre L, Casin I, Dublanchet A, Collatz E and The College de Bacteriologie-Virologie-Hygiene des Hôpitaux Généraux Français. Surveillance de la sensibilité aux antibiotiques des salmonelles et shigelles isolées dans 77 hôpitaux français. Bulletin Epidémiologique Hebdomadaire 1998;51:219-21.
24. Schmit JL, Leclercq R, Scheimberg A, Landauer D. Approche épidemiologique et clinique des entérocoques: résultat d'une enquête. Medecine et Maladies Infectieuses 1994;24S:141-8.

25. Boisivon A, Thibault M, Leclercq R, and The College de Bacteriologie-Virologie-Hygiene des Hôpitaux Généraux Français. Colonization by vancomycin-resistant enterococci of the intestinal tract of patients in intensive care units from French general hospitals. Clin Microb Infect 1997;3:175-9.
26. Perrier-Gros-Claude JD, Courrier PL, Breard JM, Vignot JL, Masseront T, Garin D, et al. Entérocoques résistants aux glycopeptides dans les viandes. Bulletin Epidémiologique Hebdomadaire1998;12:50-1.
27. Breuil J, Podglajen I, Collatz E. Susceptibility testing of anaerobic pathogens: rational and results. Proceedings of the 38th Interscience Conference on Antimicrobial Agents and Chemotherapy; 1998 Sep 24-27; San Diego, California. Washington: American Society for Microbiology; 1998. p. 636.
28. Reysset G, Trinh S, Carlier JP, Sebald M. Bases génétiques de la résistance aux 5-nitroimidazoles des Bacteroides spp. Medecine et Maladies Infectieuses 1996;26 Suppl:1-7.
29. National network on gonococcal infections. Les gonococcies en France en 1997, le réseau RENAGO. Bulletin Epidémiologique Annuel 1998 report. France: National Institute for Public Health Surveillance. In press 1999.
